# Genetic Disruption of Cilia-Associated Signaling Pathways in Patients with VACTERL Association

**DOI:** 10.3390/children10050882

**Published:** 2023-05-14

**Authors:** Jessica Ritter, Kristina Lisec, Marina Klinner, Martina Heinrich, Dietrich von Schweinitz, Roland Kappler, Jochen Hubertus

**Affiliations:** 1 Department of Pediatric Surgery, Dr. von Hauner Children’s Hospital, LMU Munich University, 80337 Munich, Germany; kristina.lisec@med.uni-muenchen.de (K.L.); jochen.hubertus@elisabethgruppe.de (J.H.); 2 Department of Diagnostic and Interventional Radiology, Klinikum Rechts der Isar, Technical University of Munich, Ismaninger Straße 22, 81675 Munich, Germany; 3 Department of Pediatric Surgery, Marien Hospital Witten, Ruhr-University Bochum, 58452 Witten, Germany

**Keywords:** VACTERL association, VATER association, whole-exome sequencing, cilia-associated signaling, sonic-hedgehog signaling pathway, wnt signaling pathway

## Abstract

VACTERL association is a rare malformation complex consisting of vertebral defects, anorectal malformation, cardiovascular defects, tracheoesophageal fistulae with esophageal atresia, renal malformation, and limb anomalies. According to current knowledge, VACTERL is based on a multifactorial pathogenesis including genomic alterations. This study aimed to improve the understanding of the genetic mechanisms in the development of VACTERL by investigating the genetic background with a focus on signaling pathways and cilia function. The study was designed as genetic association study. For this, whole-exome sequencing with subsequent functional enrichment analyses was performed for 21 patients with VACTERL or a VACTERL-like phenotype. In addition, whole-exome sequencing was performed for three pairs of parents and Sanger-sequencing was performed for ten pairs of parents. Analysis of the WES-data revealed genetic alteration in the Shh- and Wnt-signaling pathways. Additional performed functional enrichment analysis identified an overrepresentation of the cilia, including 47 affected ciliary genes with clustering in the *DNAH* gene family and the IFT-complex. The examination of the parents showed that most of the genetic changes were inherited. In summary, this study indicates three genetically determined damage mechanisms for VACTERL with the potential to influence each other, namely Shh- and Wnt-signaling pathway disruption, structural cilia defects and disruption of the ciliary signal transduction.

## 1. Introduction

The VACTERL association is a rare malformation complex consisting of vertebral defects, anorectal malformation, cardiovascular defects, tracheoesophageal fistulae with esophageal atresia, renal malformation, and limb anomalies [1,2,3]. The incidence rate is approximately 1.6:10,000 [4]. Diagnosis is based on the presence of at least three component features [5], while the appearance of only two features is defined as a VACTERL-like phenotype [4]. Today, a multifactorial event is assumed to be the cause of VACTERL [6]. Recent studies have shown several genomic alterations associated with the disorder [5,7,8,9,10,11,12]. Moreover, Kim et al. [13] demonstrated that the *Shh −/−, Gli2 −/−; Gli3 +/−* mutant mice have different combinations of a VACTERL-phenotype and postulated that Sonic Hedgehog (Shh) signaling might be central to the pathogenesis of the disorder. Further, the VACTERL mimicking *Intraflagellar Transport 172 (Ift172)^avc1^* mutant mice, showed an association with ciliopathies [14,15,16]. Primary cilia are microtubule-based, antenna-like cell organelles on the apical surface of most somatic cells [16,17]. They are important for normal cell signaling in human development and homeostasis. Besides the Shh-signaling pathway, numerous other signaling pathways such as Wingless/Int-1 (Wnt), Hippo and Notch have already been associated with the primary cilia [16,18]. Defects of the primary cilia are related to hereditary developmental diseases called ciliopathies, where some, e.g. Bardet–Biedl syndrome, may present VACTERL-like phenotypes [14,16,19]. Another type of cilia, the motile cilia, has the ability to generate fluid streams through their cilia beat. These fluid streams determine the left–right body axis and can thus influence embryonic development [20,21]. Cilia have a specific transport system, the intraflagellar transport (IFT). The IFT serves to build and maintain the cilia and therefore plays an important role in human development [15,18,22]. It consists of the complexes IFT-A, IFT-B and the BBSome, as well as the motor proteins kinesin and dynein [14,18,21]. Some skeletal ciliopathies, like Jeune syndrome, have been associated with variants of the IFT complex [17,23]. Based on the similarity between VACTERL and human ciliopathies, Hilger et al. [7] presumed a relation between variants in ciliary genes and VACTERL association. The combination of damaged Shh- and Wnt/planar cell polarity (PCP)-signaling, as well as cilia signaling was previously described by Kim et al. [24] as oligogenic event in patients with holoprosencephaly, a severe midline defect. It is unknown yet, if the VACTERL association might follow a similar genetic mechanism.

The aim of this study was to improve the understanding of the genetic mechanisms in the development of the VACTERL association. By investigating the genetic background, we aimed to transfer the known damage mechanisms in Shh-signaling and cilia-function from mouse to human, and to investigate for further associated signaling pathways and complex interrelations in the function of affected genes.

## 2. Materials and Methods

### 2.1. Patients and Basic Data Set

The current study relies on the Whole-Exome sequencing (WES) data from the work of Ritter et al. [25]. WES-Data were collected from the deoxyribonucleic acid (DNA) of 21 patients with VACTERL association or a VACTERL-like phenotype, as well as from three pair of parents (VCK1, VCK2, VCK6). The WES was performed on a HiSeq 2500 system (Illumina, San Diego, CA, USA) as described before [25]. For WES and Sanger-sequencing genomic DNA extracted from whole-blood and saliva samples was used. Whole-blood samples were collected from 24 patients with VACTERL association or a VACTERL-like phenotype, as well as from three pair of parents (VCK1, VCK2, VCK6). Additionally, saliva samples were collected from all patients’ parents by using the ORAgene-DNA OG-500 Kit (DNA Genotek, Ottawa, ON, Canada), except patient VCK10. Extraction of genomic DNA from whole-blood samples was done by using the DNeasy Blood & Tissue Kit (Qiagen, Hilden, Germany), while for saliva samples by using the prepIT-L2P Kit for DNA extraction (DNA Genotek, Ottawa, ON, Canada). Patients whose DNA samples were of poor quality and/or quantity and patients whose parents did not consent to their participation in the study were excluded. Written informed consent was obtained from each patient or, respectively, the parents. The phenotype of the patients and parents was obtained primary by live interview along with the patient records. In addition, a telephone interview was conducted to find out about any further malformations present in the family. Patients were classified as VACTERL phenotypes if presenting with at least three component features, otherwise they were classified as VACTERL-like phenotypes [4,5]. The study was approved (approval no. 026-13) by the institutional ethics committee and performed between June 2013 and September 2021.

### 2.2. Analysis of Protein Variation Effect and Gene Function

Similar to our previous publication [25], the impact of missense-typed SNVs on protein biological function was evaluated using Polymorphism Phenotyping v2 (PolyPhen-2; http://genetics.bwh.harvard.edu/pph2/index.shtml (accessed on 9 July 2020)) [26]. Additional evaluation was performed using Sorting Intolerant from Tolerant (SIFT; http://provean.jcvi.org/index.php (accessed on 28 October 2019)), and Protein Variation Effect Analyzer (PROVEAN; http://provean.jcvi.org/index.php (accessed on 28 October 2019)) databases [27,28,29]. Genome Aggregation Database (gnomAD v2.1.1; https://gnomad.broadinstitute.org (accessed on 25 January 2021)) [30] was used to obtain the allele frequencies of all damaging classified variants. These allele frequencies were checked for single nucleotide polymorphism (SNPs) with a minor allele frequency (MAF) cut off at 1% [31,32].

Identification of genes that are part of Shh- and Wnt-signaling was performed using the Kyoto Encyclopedia of Genes and Genomes (KEGG) Pathway (https://www.genome.jp/kegg/pathway.html (accessed on 20 October 2019)) [33] and Reactome (https://reactome.org/ (accessed on 9 June 2020)) databases [34,35,36,37,38]. Information on whether a gene has activating or inactivating function within its pathway was obtained from the databases GeneCards® (http://www.genecards.org/ (accessed on 17 November 2019)) [39], UniProt (https://www.uniprot.org/ (accessed on 16 October 2019)) [40] as well as Gene (https://www.ncbi.nlm.nih.gov/gene (accessed on 19 February 2022)) and PubMed® (https://www.ncbi.nlm.nih.gov/pubmed/ (accessed on 19 February 2022)), both by U.S. National Library of Medicine [41].

For functional gene enrichment analysis, the online analysis program “Database for Annotation, Visualization and Integrated Discovery (DAVID) v6.8” (https://david.ncifcrf.gov/ (accessed on 17 August 2019)) [42,43] was used as described before [25]. The main focus was set on the Gene ontology (GO) categories “biological process”, “cellular component” and “molecular function” [44,45].

### 2.3. Sanger Sequencing

Sanger sequencing was used to confirm the detected variants and to investigate their inheritance. Polymerase chain reaction (PCR) amplification primers were designed using either the Helmholtz Center Munich primer design tool or Primer-BLAST (https://www.ncbi.nlm.nih.gov/tools/primer-blast/ (accessed on 9 June 2020)) [46] (Table S1 in Supplemental Material). PCR amplification was performed with 100 ng of DNA in 1 µL of distilled H2O, 1 µL of 10 mM forward and reverse primers, and 17 µL of master mix [0.2 µL of Maxima Hot Start Taq DNA Polymerase, 2 µL of Hot Start PCR Buffer (Thermo Scientific, Waltham, MA, USA), 13.2 µL of distilled H_2_O, 1.2 µL of MgCl_2_, and 0.4 µL of 10 mM dNTPs] in a total volume of 20 μL. PCR reactions were performed on a Mastercycler Personal (Eppendorf, Hamburg, Germany) under the following conditions: initial denaturation at 95 °C for 4 min, followed by 38 cycles of denaturation at 95 °C for 40 s, annealing at 59 °C for 40 s, elongation at 72 °C for 1 min, and final elongation at 72 °C for 10 min. DNA amplicon visualization was performed using 1% agarose gel electrophoresis and a Gel Jet Imager (Intas Science Imaging Instruments, Göttingen, Germany). Purification of PCR amplicons was performed with ExoSAP-IT (Affymetrix, Santa Clara, CA, USA). Sanger sequencing was performed on a 3730 48-Capillary Array DNA Analyzer (Applied Biosystems, Foster City, CA, USA) by the LMU Munich Sequencing Service (Biocenter, Martinsried, Germany; http://www.genetik.bio.lmu.de/service/genomicsserviceunit/sequencing_service/ (accessed on 8 September 2021)) using the BigDye Terminator Cycle Sequencing Kit (Applied Biosystems, Foster City, CA, USA). Sequence analysis was performed using Chromas (Technelysium Pty Ltd., South Brisbane, Australia).

### 2.4. Literature Research and Illustrations

The database PubMed® [41] was used for literature research, especially for investigation on the gene‘s background information, including gene family, function and associated diseases. As keywords ‘VACTERL’, ‘VACTERL association’, the official gene name itself, as well as the official gene name and ‘cilia’/’ciliopathy’ were taken, the databases UniProt [40] and Genecards [39] were used to define and localize genes associated to cilia. The descriptive statistics were performed with Microsoft Excel® (Microsoft Corporation, Redmond, WA, USA). For illustrations Inkscape v.1.0 (https://inkscape.org/ (accessed on 5 June 2022)), Microsoft Excel® and Microsoft PowerPoint® (Version Microsoft Office Home and Student 2016) were used.

## 3. Results

Three patients were excluded from the study due to insufficient DNA samples. Twenty-one patients met the inclusion criteria. Their median age was 10.8 years (range: 1–31 years). The female-to-male ratio was 1:1.6 (Table 1).

### 3.1. Whole-Exome Sequencing

WES identified 3818 genetic variants, consisting of 3452 missense, 123 frameshift, 109 indel, 86 nonsense, 41 splice site and 7 stoploss variants (WES dataset VACTERL in Mendeley Data repository, https://data.mendeley.com/datasets/xkbb8wrmv4/1 (accessed on 12 July 2022)). A total of 2531 missense variants were excluded from the analysis due to their categorization as benign or possibly damaging in PolyPhen-2, SIFT, and PROVEAN scoring, resulting in 921 remaining damaging missense variants. No SNPs (MAF > 1%) were identified in the cluster of these damaging missense variants, as well as nonsense and frameshift variants (Tables S2–S4 in Supplemental Material). The average mutation rate per patient was 53.8 (range: 35–93) damaging genetic variants. In total 1130 genetic variants, including frameshift, nonsense and damaging missense variants were grouped as damaging and permitted for further investigations.

### 3.2. Signaling Pathways

First, the damaging genetic variants were screened for gene families and genes previously associated with VACTERL. Here, a *GLI1* frameshift and a *IFT172* missense variant were identified. Thereupon, we screened for signaling pathways associated with VACTERL and holoprosencephaly.

#### 3.2.1. Sonic Hedgehog Pathway

The Shh-pathway is known to play a role in the VACTERL association and is involved in embryonic development [47,48]. A total of 167 genes encoding components of Shh-signaling were identified by Reactome and KEGG. Besides *GLI1* and *IFT172*, another eight genes were affected (Table 2; Table S2 in Supplemental Material). In patient VCK1 even two damaging variants (*IFT172, PTCH2*) of Shh-signaling were detected. In total, 10 patients (48%) were affected by damaging genetic alteration in the Shh-pathway.

#### 3.2.2. Wingless/Int-1 Pathway

The Wnt-pathway is also involved in embryonic development [65] and has previously been associated with holoprosencephaly [24]. A total of 356 genes encoding components of Wnt-signaling were identified by Reactome, KEGG and literature research [66]. Hereof 14 genes were altered (Table 2; Table S3 in Supplemental Material). Of note, five patients were affected by a genetic variant within the FAT gene family. In total, 13 patients (62%) had damaging variants in the Wnt-pathway.

#### 3.2.3. Wnt and Shh Pathways

Based on the model of an oligogenic event in holoprosencephaly by Kim et al. [24], we investigated the patients for variants in the Shh- and Wnt-pathways. Here, six (29%) patients showed genetic alterations in both signaling pathways at the same time (Table 2).

### 3.3. Functional Enrichment Analysis

To investigate for further pathways and biological processes involved in VACTERL we performed an enrichment analysis with DAVID v6.8. We identified an overrepresentation of cilia in all three investigated GoTerm categories.

**Figure 1:**
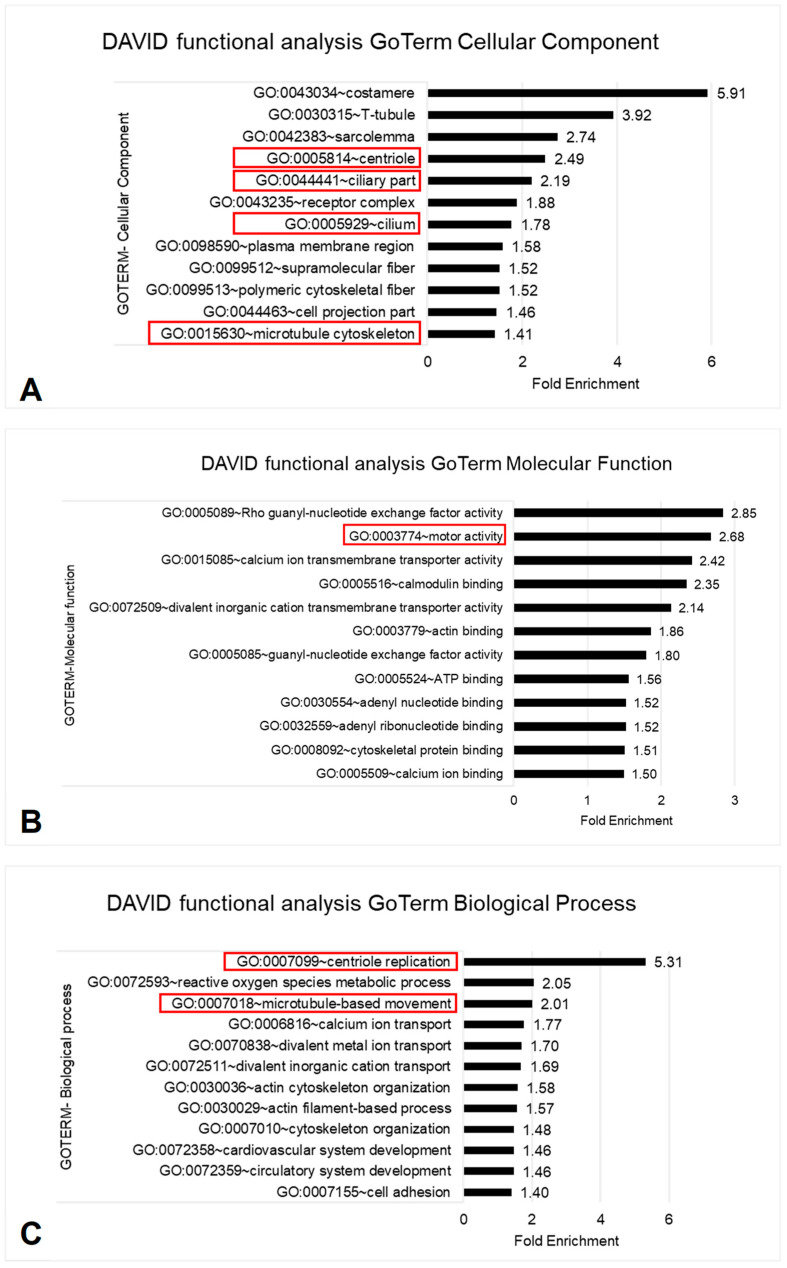
Overview top-matches in DAVID functional analysis, cilia-associated categories are highlighted red. (**A**) GoTerm Cellular Component (CC); (**B**) GoTerm Molecular Function (MF); (**C**) GoTerm Biological Process (BP).

**Figure 2:**
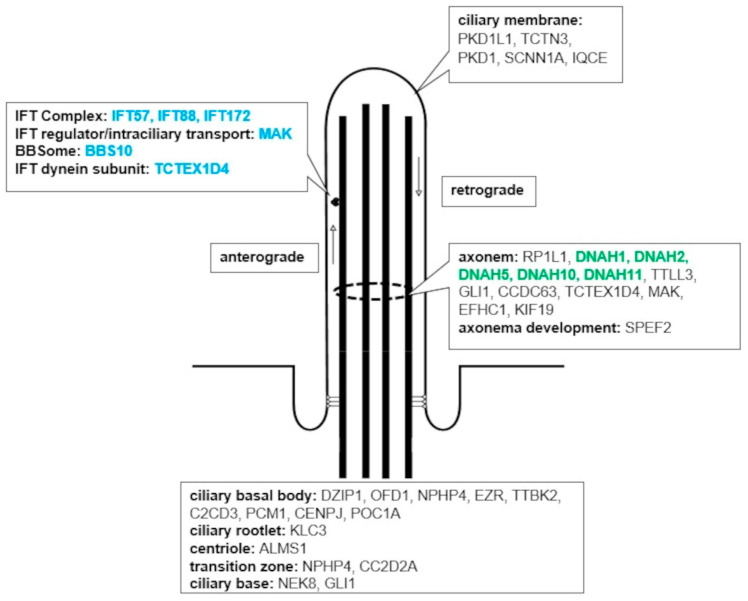
Schematic drawing of a cilia: genes coding for the different cilia parts are listed *(data obtained from Uniprot database)*; blue highlighted genes are part of the intraflagellar transport, green highlighted genes are part of the *DNAH*-gene family.

The terms centriole, ciliary part, cilium and microtubule cytoskeleton are cellular components (CC) of cilia. We have seen a fold enrichment (FE) for centriole of 2.5, for ciliary part of 2.2, for cilium of 1.8, and for microtubule cytoskeleton of 1.4 (random expectation of FE = 1.0) (Figure 1A). For the terms cilium and ciliary part DAVID functional analysis revealed 47 ciliary genes. Each of the 21 patients showed at least one as damaging classified variant in a ciliary gene (Table S4 in Supplemental Material). According to the UniProt database, gene products of 36 out of the 47 cilia genes are relevant for structure or function of the cilium (Figure 2).

In the categories of molecular function (MF) and biological process (BP) we found cilia-associated properties. The term motor activity in the category MF showed a FE of 2.7 (Figure 1B). From our ciliary genes, seven (*DNAH1, DNAH2, DNAH5, DNAH10, DNAH11, KIF19, KLC3*) were associated to the term motor activity affecting nine patients.

The terms centriole replication (FE 5.3) and microtubule-based movement (FE 2.0) represent BP (Figure 1C). From our ciliary genes, three (*C2CD3, CENPJ, OFD1*) were associated to the centriole replication affecting three patients, while twelve (*CABYR, CCDC63, DNAH1, DNAH2, DNAH5, DNAH10, DNAH11, IFT57, KIF19, KLC3, OFD1, PCM1*) were associated to the microtubule-based movement affecting twelve patients.

From the 47 cilia-associated genes, two groups of genes are remarkable. The first is represented by the genes associated with *Dynein heavy chain (DNAH*), which includes the genes *DNAH1, DNAH2, DNAH5, DNAH10, DNAH11*, and affected nine (43%) patients (Table S4 in Supplemental Material). Of note, the siblings VCK3 and VCK16 showed the same damaging variant in *DNAH2*. The other group is represented by the IFT complex. Variants in genes coding for the IFT, including *BBS10, IFT57, IFT88, IFT172, MAK* and *TCTEX1D4* affected five (24%) patients (Table S4 in Supplemental Material).

### 3.4. Inheritance

**Figure 3:**
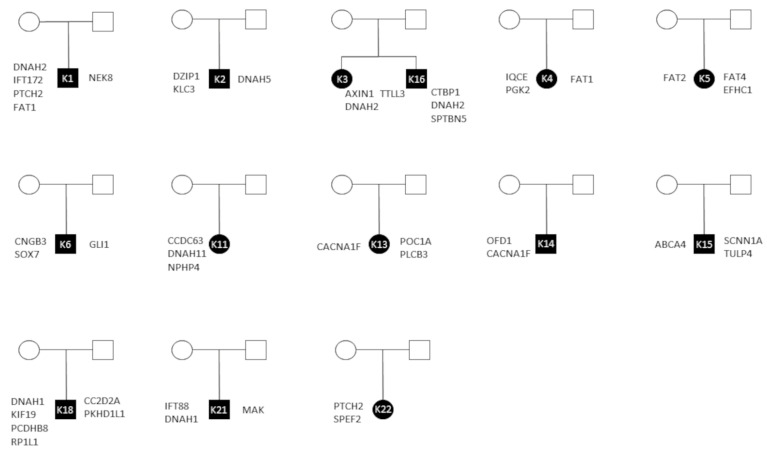
Pedigrees: black filled = diseased; right sided genes are inherited by father, left sided genes are inherited by mother.

Further, we analyzed triplets of the revealed genetic variants from both, cilia and signaling pathways by Sanger sequencing of the parents. Seven families were excluded due to missing DNA or DNA of poor quality (VCK10, VCK17, VCK20), the related patient only showing one cilia gene variant and no signaling pathway variants altered (VCK7, VCK8, VCK9) and insufficient Sanger sequences (VCK19). In total, 13 pair of parents were examined successful. Most (46 from 72; 64%) genetic variants were inherited. Inheritance for signaling pathway genes and ciliary genes by both parents was seen in ten patients (Figure 3). Here, we noticed, that the parents themselves inherited damaging variants, but had no history of malformation. The children, however, showed a combination of the inherited damaging variants. Four children showed inheritance of their genetic variants by only one parent (Figure 3), while in one case (VCK14) an X-chromosomal inherited frameshift variant in *OFD1* was observed [49]. The variants in *GNAT2* (VCK15) and *TCTN3* (VCK18) were found to be de novo.

## 4. Discussion

As previous studies suggested that VACTERL association is based on a multifactorial pathogenesis including genomic alterations [6], WES and functional enrichment analyses were performed in our study.

First, we focused on variants affecting genes of the Shh- and Wnt-pathways. This approach relies on several genetic alterations of the Shh-pathway that have been identified in previous studies in patients with VACTERL association [6], and the VACTERL-mimicking mice models by Kim et al. [13] affecting the *Gli* gene family, which is central to the Shh-pathway. We identified ten patients (48%), almost half of our patient cohort, that were affected by a genetic alteration in Shh-signaling, whereas one patient was found to have a damaging *GLI1* variant. This may indicate that in humans, as already shown in mice models, a disruption of the Shh-signaling pathway is associated with VACTERL phenotypes. This signaling disruption may be caused by alteration of activating components, as our results show half of the Shh-signaling-associated genes as having activation function and only *PTCH2* working to inactivate (Table 2) [49,50,51,52,53,54,55]. The impairment of Shh-signaling by alteration of activating components is in line with the findings of Kim et al. [47], who reported a lack of Shh-signaling leading to severe disruption of the somite patterning with consecutive vertebral defects. The general downregulation along with the heterogenous single gene variants led us thought of the VACTERL association to be more caused by a general disruption of the signaling cascade, than by a single gene mutation.

The Wnt-pathway was associated with holoprosencephaly [24], playing a central role in embryonic development [65]. Genetic alterations of the Wnt-signaling was found in more than half of the patient cohort (62%, 13 patients). In contrast to Shh-signaling, mainly inactivating gene products were detected (Table 2) [56,57,58,59,60,61,62,63]. However, similar to the Shh-pathway, we assume a general disruption of the regular signaling cascade. Our theory is strengthened by the review from Grigoryan et al. [67] and Miyagawa and Harada et al. [68], who demonstrated that both over- and under-expression of the Wnt-signaling pathway, respectively, with beta-catenin as the central molecule of Wnt-signaling, can lead to phenotypic changes in mice. Additionally, Miyagawa and Harada et al. [68] demonstrated that the Shh-signaling pathway and beta-catenin mutually influence each other’s expression. Following this reciprocal influence, gene variants in both pathways were found in one-third of our patients.

Second, we investigated variants affecting genes, which are associated with cilia structure and functioning. Recent literature has shown similarities between ciliopathies and VACTERL. So, ciliary defects can lead to cardiac and renal defects or malformation of the extremities, giving ciliopathies a VACTERL-like appearance [14,16,19]. Our functional enrichment analysis revealed an overrepresentation of mutated genes coding for components of the cilia. All 21 patients showed genetic alteration in a ciliary gene, indicating a structural cilia defect associated with VACTERL-association. Further, more than half (57%, 12 patients) of our patients showed genetic variants associated with the microtubule-based movement of cilia, suggesting an additional defect of ciliary movement in patients with VACTERL-association.

A closer look on the cilia genes revealed clustering in the *DNAH* gene family (43%, nine patients) and in the IFT complex (24%, five patients). The *Ift172^avc1^* mutant mouse has already been associated with VACTERL as well as Shh-signaling, the IFT-complex, and ciliary defects in general [14]. Within these mouse embryos the *Ift172* mutation induced a global reduction in the expression of *Ptch1*, another gene of the Shh-signaling pathway [14]. Similar molecular processes could take place in our patient VCK1, who showed a *PTCH2* variant, a paralog to *PTCH1* with related function [69], in addition to an *IFT172* mutation. This supports our theory introduced above, that single gene mutation may cause signaling pathway impairment, in this case by global reduction in expression of genes associated with the same pathway. Further, the researchers found structural, ciliary defects in the mouse embryos, also caused by a mutation-induced reduction of *Ift172* expression. These findings are in line with our detection of altered genes associated with cilia structure as described previously. The *DNAH* gene family codes for chain proteins of the microtubule-associated motor protein dynein, which is found in cilia and flagella. Their gene products play an essential role for correct cilia function, including cilia motility [70,71,72,73,74,75]. Both, the IFT complex and the motor protein dynein play an important role in the signal transduction of embryological signaling pathways [16,17,18]. They work together along the axoneme, a structural skeleton in the center of primary cilia built from microtubules [17,18]. Therefore, we assume a general disruption of the signal transduction, caused by structural cilia and ciliary movement defects, may be seen in patients with VACTERL association. In addition, cilia are known to control signaling pathway activity. So, cilia can both activate and inactivate the Shh-signaling pathway and can also lead to a reduction of the Wnt-signaling pathway [16,18].

Third, we investigated the inheritance of the signaling pathway and the cilia-associated genetic variants. The majority was inherited, in most patients (10 of 14 patients) even by both parents. The inheritance shows different compositions of the three damage mechanisms: signaling pathway disruption, structural cilia defect and disruption of the signal transduction. We assume the combination of these different damage mechanisms leading to the formation of complex malformations and to phenotypes of varying severity. The theory of heterogeneous malformation syndromes caused by a combination of different damage mechanisms, including disruption of the primary cilia, Shh- and Wnt/PCP-signaling pathways, was previously described by Kim et al. [24] as an oligogenic event in patients with holoprosencephaly.

The limitation of the study is its small number of patients with a total of 21 included patients. This is due to the rarity of the VACTERL association and the study design with only in-house patient recruitment. Accordingly, it is important that cell and animal models are realized as a next step to further investigate these molecular interactions during the development of VACTERL.

## 5. Conclusions

In summary, this study indicates three different damage mechanisms for VACTERL association. The first damage mechanism is the general disruption of Shh- and Wnt-signaling pathways, known to mutually influence each other’s activity. Our results suggest a general downregulation of Shh-signaling by the genetic alteration of activating components, as well as an activation of the Wnt-signaling pathway by genetic alteration of inactivating components. The second and third damage mechanism affect the cilia structure or the ciliary signal transduction, respectively. We suggest these may result in a general disruption of the cilia function, as well as loss of control of the signaling pathway activity for the Shh- and Wnt-signaling pathways. All three mechanisms have been individually associated with VACTERL-like diseases or mouse models showing VACTERL(-like) phenotypes before. Interestingly, our patients showed different combinations of the three damage mechanisms, which may arise in part from the inheritance of genetic changes from patients’ mothers and fathers. Additionally, all three damaging mechanisms show the potential to influence each other, but further studies are needed.

## Figures and Tables

**Table 1:** Overview of the demographic data and disease specifications in different patient subgroups. The table shows the total number of VACTERL criteria met, as well as their distribution within the overall cohort and within different gene families and pathway groups. In addition, a distribution of other malformations across all patient groups is listed. The percentage always refers to the total number of 21 patients.

	All	Wnt-Group	Shh-Group	Wnt/Shh-Group	*DNAH*-Group	IFT-Group
	n (%)	n (%)	n (%)	n (%)	n (%)	n (%)
Total	21 (100)	13 (62)	10 (48)	6 (29)	9 (43)	5 (24)
**Gender**						
Female	8 (38)	5 (24)	3 (14)	2 (10)	2 (10)	1 (5)
Male	13 (62)	8 (38)	7 (33)	4 (19)	7 (33)	4 (19)
Female-to-male ratio	1:1.6	1:1.6	1:2.3	1:2.0	1:3.5	1:4.0
**Age at the end of data acquisition (December 2019)** Years, median (range)	10.8 (1–31)	11.1 (1–31)	9.3 (2–19)	9.7 (3–19)	8.2 (1–16)	11.2 (5–17)
**Fulfilled VACTERL criteria**						
2	1 (5)	0 (0)	1 (5)	0 (0)	1 (5)	1 (5)
3	7 (33)	4 (19)	5 (24)	3 (14)	4 (19)	2 (10)
4	4 (19)	4 (19)	0 (0)	0 (0)	2 (10)	0 (0)
5	8 (38)	5 (24)	3 (14)	3 (14)	1 (5)	2 (10)
6	1 (5)	0 (0)	1 (5)	0 (0)	1 (5)	0 (0)
**Distribution of VACTERL criteria**						
Vertebral anomalies	17 (81)	10 (48)	7 (33)	4 (19)	6 (29)	3 (14)
Anorectal malformation	12 (57)	8 (38)	6 (29)	5 (24)	5 (24)	3 (14)
Cardiac defects	17 (81)	12 (57)	7 (33)	5 (24)	7 (33)	3 (14)
Tracheoesophageal defects	16 (76)	10 (48)	9 (43)	5 (24)	6 (29)	5 (24)
Renal anomalies	15 (71)	8 (38)	7 (33)	4 (19)	7 (33)	4 (19)
Limb anomalies	8 (38)	5 (24)	2 (10)	1 (5)	2 (10)	0 (0)
**Distribution of other anomalies**						
Other gastro-intestinal anomalies	9 (43)	5 (24)	5 (24)	3 (14)	3 (14)	1 (5)
Oro-facio-auricolo anomalies	8 (38)	4 (19)	4 (19)	1 (5)	3 (14)	1 (5)
Genital anomalies	8 (38)	4 (19)	4 (19)	2 (10)	6 (29)	3 (14)

**Table 2:** Overview on patients’ phenotype (V-vertebral column, A-anorectum, C-cardiovascular system, TE-trachea and esophagus, R-urinary tract, L-limbs) and altered Shh- and Wnt-signaling pathway genes. Blue highlighted: patients with damaging variant in both pathways. Green highlighted: Activating genes; Red highlighted: Inactivating genes. Grey highlighted: patients did not show genetic variants in at least one of the signaling pathways.

Patient	Gender	Fulfilled VACTERL Criteria	Shh [49,50,51,52,53,54,55]	Wnt [56,57,58,59,60,61,62,63,64]
VCK1	M	V, A, C, TE, R	*PTCH2, IFT172*	*FAT1*
VCK2	M	V, A, C, TE, R, L	*DZIP1*	
VCK3	F	V, A, C, R		*AXIN1*
VCK4	F	V, A, C, TE, L	*IQCE*	*FAT1*
VCK5	F	V, A, C, TE, R		*FAT2, FAT4*
VCK6	M	V, A, C, TE, R	*GLI1*	*SOX7*
VCK7	F	V, A, C, R, L		
VCK8	M	V, A, C, TE, R		
VCK9	M	V, C, TE, R, L		
VCK10	M	V, C, TE, L		*KREMEN2, AP2A2*
VCK11	F	V, A, R		
VCK13	F	V, C, TE, L		*PLCB3*
VCK14	M	V, A, C	*OFD1*	*TLE5*
VCK15	M	V, C, TE, R, L		*GNAT2*
VCK16	M	V, A, C, R		*CTBP1*
VCK17	F	A, TE, R	*IFT57*	*FAT4*
VCK18	M	C, TE, L		*LRP5*
VCK19	M	C, TE, R	*LRP2*	
VCK20	M	C, TE, R	*CSNK1G1*	*FAT4, APC2, MMP7*
VCK21	M	V, TE	*IFT88*	
VCK22	F	V, TE, R	*PTCH2*	

## Data Availability

Whole-exome-sequencing dataset for all patients will be available with publication. The dataset WES dataset VACTERL for this study can be found in Mendeley Data https://data.mendeley.com/datasets/xkbb8wrmv4/1 (accessed on 12 July 2022).

## Supplementary Materials

The following supporting information can be downloaded at: https://www.mdpi.com/article/10.3390/children10050882/s1, Table S1: Forward and reverse primers sequences and additional data for sanger sequenced genes, Table S2: Genetic variants in Shh-signaling pathway genes with predicted damaging potential in patient cohort, “-” shows no prediction was possible because of the frameshift or nonsense character of the genetic variant, Table S3: Genetic variants in Wnt-signaling pathway genes with predicted damaging potential in patient cohort, “-” shows no prediction was possible because of the frameshift or nonsense character of the genetic variant, Table S4: Genetic variants in ciliary genes with predicted damaging potential in patient cohort, “-” shows no prediction was possible because of the frameshift or nonsense character of the genetic variant.
