# Supplementary material for: Genetic Disruption of Cilia-Associated Signaling Pathways in Patients with VACTERL Association
Source: Children (Basel). 2023 May 14;10(5):882. doi: 10.3390/children10050882 (PMC10217539; doi:10.3390/children10050882)
Supplement: Supplementary file 1 [file children-10-00882-s001.zip › children-2378361-supplementary.pdf]

Supplementary Material

Supplementary tables:

Table S1

Table S2

Table S3

Table S4

Table S1. Forward and reverse primers sequences and additional data for sanger sequenced genes.

Patient	Gene	Sequenz Primer Forward	GC %	Sequenz Primer Reverse	GC %	Product size	Annealing Temp in °C
VCK 1	*FAT1*	ACATGACTTTCGTTCATGGTTCT	39.10	GGACCAAATGGTGGGAGGAG	60.00	398	59
VCK 4	*FAT1*	GCTGGCGTATGCTACAGAGG	60.00	TGGACAGAATTTGTAACCTCACTG	41.70	750	60
VCK 6	*GLI1*	GGAACCTACAGCCAGTGTCC	60.00	TGCGATCTGTGATGGATGAGA	47.60	962	59
VCK 3	*AXIN1*	CCTCAGCACACGCTGTACG	63.16	GTGTCACAAGCTGCCTCCC	63.16	139	60
VCK 3	*DNAH2*	TCATCACCCACCATCTACTCAG	50.00	CGCCGTTGATGAGGGTC	64.71	136	60
VCK 4	*IQCE*	GGTTCAGTGTCTCTTTTCTGGG	50.00	TGTGATCTGACTGGCTCTGC	55.00	139	60
VCK 4	*PGK2*	ATTGACAGGACCATGAGCCA	50.00	CGGATGCTTTATGGGGCAGA	55.00	761	60
VCK 5	*FAT2*	CCGTCTCCATGACCGTAAAG	55.00	ATCAAGGCAACAGACAGTGG	50.00	139	60
VCK 5	*FAT4*	TGATAACAACCCCATCTTTGC	42.86	CACCTGTGACAGAGTCTATCCG	54.55	185	60
VCK 5	*CATSPERG*	ACACGAGCACCTACTGCAAC	55.00	CCCACCCAGTAGGGCAG	70.59	128	60
VCK 5	*EFHC1*	GCCAAGTTGAACTCCCTACTTTTAG	44.00	GATAGTAATGAATGATGTAGGTCCG	40.00	137	60
VCK 10	*KREMEN2*	GCAGTAACCCAGACGGTGAC	60.00	GTTCCCACCTCTGTCCAGC	63.16	127	60
VCK 10	*AP2A2*	GTCTCATTGCCTGTGCTGTC	55.00	GTGCAGCTTCAGGAGCC	64.71	168	60
VCK 10	*EZR*	TCCTGACTTGCCTTCTTATCC	47.62	CTCTCTCTCTCACGCAGGTG	60.00	124	60
VCK 11	*CCDC63*	CTGCTGAAGCTGGCTGAGAG	60.00	TCTCCATGTCGTTGTTGAGC	50.00	118	60
VCK 11	*DNAH11*	TCAGAAATTCTTGAGAAACCCC	40.91	CCATAATCAATATGCTGCCG	45.00	164	60
VCK 11	*NPHP4*	CACACGAGCCAGGAGGC	70.59	CAAAGGTGTCTTCGTGCTGC	55.00	141	60
VCK 13	*PLCB3*	TTCTATACCCAGCCAGAGCC	55.00	CAGGACCTGCTGCTGCC	70.59	138	60
VCK 13	*CACNA1F*	AACCGGATCCCAGGAAGTAG	55.00	TTCCATCATGAAGGCTCTGG	50.00	137	60
VCK 13	*POC1A*	GATGACGGGTGAACCCAC	61.11	GCAGGAGTGTGGAGTCTGTG	60.00	140	60
VCK 14	*OFD1*	TTTAGTCTGTGGAACTAGACACAT	37.50	GCTTTCGGTCATAGGTCTCCT	52.38	196	60
VCK 14	*AES*	GAGGCTTCGGCTCCACC	70.59	tcacaagaatgGCTTCCCTC	50.00	406	60
VCK 14	*CACNA1F*	GCCCTCTTCAGCCATAGAAC	55.00	GAAGCAGCAGATGGAGGAAG	55.00	118	60
VCK 15	*GNAT2*	ATCATAGGCACTGAGGGCTG	55.00	ATGAAAATAGCTGCTTCTTCCC	40.91	129	60
VCK 15	*ABCA4*	CCAGCCCAGGAGACTGAG	66.67	AGATGCCAAGGTGGTGATTC	50.00	126	60
VCK 15	*SCNN1A*	GCTGGAGGCCACGCTAC	70.59	CAGCTCCTTCACCACTCTCG	60.00	136	60
VCK 15	*TULP4*	GTCCTCTCCCTGACCGAAAG	60.00	CCTCCTTCTTCACTTTGCCC	55.00	133	60
VCK 16	*CTBP1*	ACTGGGACAGAGGCTGCTC	63.16	ACTGTGGCCTTCTGCGAC	61.11	177	60
VCK 16	*DNAH2*	TCATCACCCACCATCTACTCAG	50.00	CGCCGTTGATGAGGGTC	64.71	136	60
VCK 16	*SPTBN5*	CCTCTTCCCAGTGGACCTG	63.16	TTCAAGGCCTGAGAAGCAAG	50.00	123	60

VCK 16	*TTLL3*	GTGGTGAGTGTGGGCAG TC	63. 16	AGTAGCAGCGTGGGAAG AAG	55. 00	130	60
VCK 17	*IFT57*	GTGTTCCACAATGCCAA TTC	45. 00	TGGGCTTAAGACAAAGTG AGG	47. 62	182	60
VCK 17	*FAT4*	AAAAGCCAATGATCAA GCTG	40. 00	CTGATGGGTCTGCAGCAA G	57. 89	134	60
VCK 17	*CABYR*	CACATCTTTGAAAGGTC AGCC	47. 62	TCCTGCCCAGTATTGTCA GAG	52. 38	135	60
VCK 17	*PKD1L 1*	GCCAAATGCAGAAGAC AAGG	50. 00	GATGCCTGTTTTGGGACA AG	50. 00	152	60
VCK 18	*LRP5*	GCCTGGCTGAGTATTTCC CTT	52. 38	CGGTCCAGTAGAGGTTTC GG	60. 00	383	60
VCK 18	*CC2D2 A*	TTTTCTTAGAGTCCTGAT CCTGTTC	40. 00	TCCATCAGACAGGTTTTG GG	50. 00	140	60
VCK 18	*DNAH1*	GGCTACTGCAGGACCAA GTG	60. 00	TCCATGCAGCGCTTGAG	58. 82	127	60
VCK 18	*KIF19*	CACTGATCCTGCCCCTTT C	57. 89	CCATCTTTGCCATGGGTG	55. 56	141	60
VCK 18	*PCDHB 8*	CTTCTCGGTGCTCCTGTT CG	60. 00	GAAAACAAGACAAATAC TATGGGCA	36. 00	414	60
VCK 18	*PKHD1 L1*	TACGGTCACACTCCCTG ATG	55. 00	TCAGCATTTCCAAATTTC CAC	38. 10	334	60
VCK 18	*RP1L1*	TACGCTACTCTCCCCTGA GC	60. 00	ACTCAGGCCTCTGGGAGC	66. 67	140	60
VCK 18	*TCTN3*	GGTTGGTTTGTCCCAAA CTG	50. 00	GTCCTAAACATATTCGGC CC	50. 00	133	60
VCK 19	*LRP2*	GTGCGTCTCGTTTTATCA GC	50. 00	CATTAGTAGGAAAATTGA TGCCTTAC	34. 62	208	60
VCK 19	*ALMS1*	GAGTCTGAAATTAGGAG AGCTGTG	45. 83	AAGCGGGTGCTGAGATA CAC	55. 00	281	60
VCK 19	*CENPJ*	CATACCACTCTTTGGTCT GGC	52. 38	TCTTAGGTGATAATCTTGT GTTGG	37. 50	178	60
VCK 19	*DNAH1 0*	CCAGAACCTTGAAAATC CACC	47. 62	CTCTTGAAATCGGAGGAT GG	50. 00	115	60
VCK 19	*PKD1*	GCGTGAAGTTGTGTGTC ACC	55. 00	ACATCACGTGGACCTTCG AC	55. 00	333	60
VCK 20	*CSNK1 G1*	AGCATTGAAACAAAGCC CTC	45. 00	CGGAGAGATGATTTGGA AGC	50. 00	120	60
VCK 20	*FAT4*	TGAAAACACACTTACTG GAACAG	39. 13	CACCTGTGACAGAGTCTA TCCG	54. 55	137	60
VCK 20	*APC2*	CACAGTCTCCCTTGTGTC CC	60. 00	GGTGGAGCTCCTCACTGT C	63. 16	130	60
VCK 20	*MMP7*	CTCATCGAAGTGAGCAT CTCC	52. 38	TTCTAGCCTCAATAATCG TATAAACTG	33. 33	190	60
VCK 20	*C2CD3*	GCCTTGATGGAACCTGA AAC	50. 00	GGGGACATTCCTTACAAT CC	50. 00	272	60
VCK 20	*DNAH5*	CTGAAATTAGGGCAGGG AAC	50. 00	CAGGTGCCTTCCTACCAA TG	55. 00	140	60
VCK 20	*DNAH1 1*	TGATAGAGGGAAGGAC CCAC	55. 00	ACATGTTGCATGGCATCT TC	45. 00	141	60
VCK 20	*SPEF2*	ATACAGCAGGCCAGAC AACC	55. 00	CATTAACAATTTCCGCCG TC	45. 00	137	60
VCK 20	*TCTEX 1D4*	TTTTGCAGAATTGGACTC CC	45. 00	TGCAGTGTGGTGCTGGG	64. 71	175	60
VCK 21	*IFT88*	TCAGAATTTTAAAGCTA ATCCATGTC	30. 77	CAGATTTGGTGCCATGCT C	52. 63	141	60
VCK 21	*DNAH1*	CCGAGTACGAGTGCTTC CAG	60. 00	AAGAGGAAGGTGATGGG TAGG	52. 38	121	60
VCK 21	*MAK*	CGGATTCATTACTCTTGC CC	50. 00	TTCAACAAGGAAGTCATT TCAATC	33. 33	132	60
VCK 22	*PTCH2*	CCTTCTACCCAGAGCTG TTCC	57. 14	GGGATCCAGAGACATTGT GG	55. 00	119	60
VCK 22	*SPEF2*	CATGTTCGGCATGAAAA GG	47. 37	CAAAATATAGCTTTGTTT ACCACG	33. 33	191	60

Table S2: Genetic variants in Shh-signalling pathway genes with predicted damaging potential in patient cohort, "-" shows no prediction was possible because of the frameshift or nonsense character of the genetic variant.

Gene	Variant	gnomAD allele frequency	PPH2	SIFT	Provean	Affected Patient
GLI1	c.3307delA p.N1103T fsTer7	~0.000006	-	-	-	VCK6
PTCH2	c.203G->T p.A68D	~0.00001	Probably damaging	Damaging	Deleterious	VCK22
	c.247C->T p.E83K	~0.00003	Probably damaging	Damaging	Deleterious	VCK1
IFT172	c.3400G->A p.R1134W	~0.0002	Probably damaging	Damaging	Deleterious	VCK1
IFT57	c.1119C->A p.L373F	~0.0001	Probably damaging	Damaging	Deleterious	VCK17
IFT88	c.884G->C p.G295A	Unknown	Probably damaging	Damaging	Deleterious	VCK21
LRP2	c.2356C->T p.V786I	~0.000008	Probably damaging	Tolerated	Neutral	VCK19
CSNK1G1	c.745G->A p.L249F	Unknown	Probably damaging	Damaging	Deleterious	VCK20
DZIP1	c.1033C->G p.D345H	Unknown	Probably damaging	Damaging	Deleterious	VCK2
IQCE	c.1045A->G p.K349E	Unknown	Probably damaging	Damaging	Deleterious	VCK4
OFD1	c.974_975insT p.E326*	Unknown	-	-	-	VCK14

Table S3: Genetic variants in Wnt-signalling pathway genes with predicted damaging potential in patient cohort, "-" shows no prediction was possible because of the frameshift or nonsense character of the genetic variant.

Gene	Variant	gnomAD allele frequency	PPH2	SIFT	Provean	Affected Patient
FAT1	c.4433 A->G p.I1478T	~0.00009	Possibly damaging	Damaging	Deleterious	VCK1
	c.12899 G->A p.A4300V	~0.00001	Probably damaging	Tolerated	Neutral	VCK4
FAT2	c.4028G->A p.S1343F	~0.000004	Probably damaging	Damaging	Deleterious	VCK5
FAT4	c.6532G->A p.A2178T	~0.0002	Possibly damaging	Damaging	Deleterious	VCK5
	c.4543G->A p.V1515M	~0.00005	Probably damaging	Damaging	Neutral	VCK17
	c.6562C->T p.Q2188Stop	Unknown	-	-	-	VCK20
APC2	c.1463G->A p.R488H	~0.0001	Probably damaging	Damaging	Deleterious	VCK20
MMP7	c.487G->A p.H163Y	~0.000004	Probably damaging	Damaging	Deleterious	VCK20
LRP5	c.1192C->T p.R398C	~0.00007	Probably damaging	Damaging	Deleterious	VCK18
CTBP1	c.204_205insA p.G69Rfs*78	~0.00002	-	-	-	VCK16
PLCB3	c.3418G->C p.E1140Q	~0.0002	Probably damaging	Tolerated	Neutral	VCK13
AXIN1	c.1378G->A p.R460W	~0.00005	Probably damaging	Damaging	Neutral	VCK3
SOX7	c.203C->T p.G68E	~0.00001	Probably damaging	Damaging	Deleterious	VCK6
KREMEN2	c.326T->G p.I109S	Unknown	Probably damaging	Damaging	Deleterious	VCK10
AP2A2	c.742A->G p.T248A	~0.000004	Probably damaging	Tolerated	Deleterious	VCK10
AES/TLE5	c.200C->T p.W67Stop	~0.00003	-	-	-	VCK14
GNAT2	c.633C->G p.W211C	Unknown	Probably damaging	Damaging	Deleterious	VCK15

Table S4: Genetic variants in ciliary genes with predicted damaging potential in patient cohort, "-" shows no prediction was possible because of the frameshift or nonsense character of the genetic variant.

Gene	Variant	gnomAD allele frequency	PPH2	SIFT	Provean	Affected Patient
ABCA4	c.3292G->A<br>p.R1098C	~0.00002	Probably damaging	Damaging	Deleterious	VCK15
ALMS1	c.770T->G<br>p.I257S	Unknown	Probably damaging	Damaging	Deleterious	VCK19
BBS10	c.1838T->C<br>p.Y613C	~0.00003	Probably damaging	Damaging	Deleterious	VCK8
C2CD3	c.519G->T<br>p.Y173Stop	~0.00002	-	-	-	VCK20
CABYR	c.941G->A<br>p.G314D	~0.000008	Probably damaging	Damaging	Neutral	VCK17
CACNA1F	c.761A->G<br>p.I254T	~0.00006	Probably damaging	Damaging	Deleterious	VCK13
	c.1234C->T<br>p.E412K	~0.00002	Probably damaging	Damaging	Deleterious	VCK14
CATSPERG	c.3193C->T<br>p.R1065W	~0.00003	Probably damaging	Damaging	Deleterious	VCK5
CC2D2A	c.3055C->T<br>p.R1019Stop	~0.0001	-	-	-	VCK18
CCDC63	c.983C->T<br>p.A328V	Unknown	Probably damaging	Damaging	Neutral	VCK11
CENPJ	c.3532G->A<br>p.R1178Stop	~0.000008	-	-	-	VCK19
CNGB3	c.1582C->A<br>p.E528Stop	~0.00001	-	-	-	VCK6
DNAH1	c.7441G->A<br>p.V2481M	Unknown	Probably damaging	Damaging	Deleterious	VCK18
	c.7865G->A<br>p.R2622Q	~0.0003	Probably damaging	Damaging	Deleterious	VCK21
DNAH2	c.4648C->T<br>p.R1550Stop	~0.000004	-	-	-	VCK1
	c.6528G->C<br>p.E2176D	~0.00003	Probably damaging	Damaging	Deleterious	VCK3, VCK16
DNAH5	c.11476G->A<br>p.L3826F	~0.00034	Probably damaging	Damaging	Deleterious	VCK2
	c.9244G->T<br>p.L3082I	~0.0006	Probably damaging	Damaging	Neutral	VCK20
DNAH10	c.1716A->G<br>p.I572M	~0.000008	Probably damaging	Damaging	Deleterious	VCK19
DNAH11	c.8452T->C<br>p.Y2818H	Unknown	Probably damaging	Tolerated	Deleterious	VCK20
	c.7729G->A	Unknown		Damaging	Deleterious	VCK11

	p.D2577N		Probably damaging			
DZIP1	c.1033C->G<br>p.D345H	Unknown	Probably damaging	Damaging	Deleterious	VCK2
EFHC1	c.737A->G<br>p.Y246C	~0.000008	Probably damaging	Damaging	Deleterious	VCK5
EZR	c.146T->C<br>p.Y49C	~0.00002	Probably damaging	Damaging	Deleterious	VCK10
GLI1	c.3307delA<br>p.N1103T fsTer7	~0.000006	-	-	-	VCK6
GNAT2	c.633C->G<br>p.W211C	Unknown	Probably damaging	Damaging	Deleterious	VCK15
IFT57	c.1119C->A<br>p.L373F	~0.0001	Probably damaging	Damaging	Deleterious	VCK17
IFT88	c.884G->C<br>p.G295A	Unknown	Probably damaging	Damaging	Deleterious	VCK21
IFT172	c.3400G->A<br>p.R1134W	~0.0002	Probably damaging	Damaging	Deleterious	VCK1
IQCE	c.1045A->G<br>p.K349E	Unknown	Probably damaging	Damaging	Deleterious	VCK4
KIF19	c.2920C->T<br>p.R974Stop	~0.00008	-	-	-	VCK18
KLC3	c.509G->A<br>p.R170H	~0.00007	Probably damaging	Tolerated	Neutral	VCK2
MAK	c.10_11insATCG<br>p.T5Rfs*21	Unknown	-	-	-	VCK21
NEK8	c.1223_1223delA<br>p.D408Afs*6	Unknown	-	-	-	VCK1
NPHP4	c.3895C->T<br>p.G1299S	~0.0001	Probably damaging	Tolerated	Deleterious	VCK11
OFD1	c.974_975insT<br>p.E326*	Unknown	-	-	-	VCK14
PCDHB8	c.2203G->A<br>p.G735R	~0.00001	Probably damaging	Damaging	Deleterious	VCK18
PCM1	c.3841A->C<br>p.K1281Q	~0.000008	Probably damaging	Damaging	Neutral	VCK9
PGK2	c.509T->A<br>p.H170L	~0.0005	Probably damaging	Damaging	Deleterious	VCK4
PKD1	c.3994C->T<br>p.D1332N	~0.0002	Probably damaging	Tolerated	Deleterious	VCK19
PKD1L1	c.5125C->G<br>p.G1709R	~0.0002	Probably damaging	Damaging	Deleterious	VCK17
PKHD1L1	c.7204T->C<br>p.W2402R	~0.001	Probably damaging	Damaging	Deleterious	VCK18
POC1A	c.1085G->A<br>p.T362M	~0.0002	Probably damaging	Damaging	Deleterious	VCK13
RP1L1	c.3569G->A	~0.0001		Damaging	Neutral	VCK18

	p.T1190M		Probably damaging			
SCNN1A	c.745G->A<br>p.R249C	~0.0001	Probably damaging	Damaging	Deleterious	VCK15
	c.744C->A<br>p.Q248H	~0.0001	Probably damaging	Tolerated	Neutral	
SPEF2	c.1159C->G<br>p.R387G	~0.00003	Probably damaging	Damaging	Deleterious	VCK22
	c.887G->A<br>p.R296H	~0.00001	Probably damaging	Damaging	Deleterious	VCK20
SPTBN5	c.4243G->C<br>p.L1415V	~0.00004	Probably damaging	Damaging	Deleterious	VCK16
TCTEX1D4	c.601A->G<br>p.S201P	~0.0001	Probably damaging	Damaging	Deleterious	VCK20
TCTN3	c.978A->C<br>p.Y326Stop	Unknown	-	-	-	VCK18
TTBK2	c.3418G->A<br>p.P1140S	~0.00006	Probably damaging	Tolerated	Neutral	VCK7
TTLL3	c.766C->T<br>p.R256W	~0.0001	Probably damaging	Damaging	Deleterious	VCK16
TULP4	c.4012C->G<br>p.R1338G	Unknown	Probably damaging	Damaging	Deleterious	VCK15
